# Health and social impacts of open defecation on women: a systematic review

**DOI:** 10.1186/s12889-019-6423-z

**Published:** 2019-02-06

**Authors:** Mahrukh Saleem, Teresa Burdett, Vanessa Heaslip

**Affiliations:** Bournemouth University, Bournemouth, Dorset UK

**Keywords:** Open defecation, Women, Low and middle income countries, Public health, Health inequalities, Social inequalities

## Abstract

**Background:**

The significance of sanitation to safeguard human health is irrefutable and has important public health dimensions. Access to sanitation has been essential for human dignity, health and well-being. Despite 15 years of conjunctive efforts under the global action plans like Millennium Development Goals (MDGs), 2.3 billion people have no access to improved sanitation facilities (flush latrine or pit latrine) and nearly 892 million of the total world’s population is still practicing open defecation.

**Methods:**

The study provides a systematic review of the published literature related to implications of open defecation that goes beyond the scope of addressing health outcomes by also investigating social outcomes associated with open defecation. The Preferred Reporting Items for Systematic reviews and Meta-Analyses (PRISMA) was used to frame the review, empirical studies focusing upon open defecation in women aged 13–50 in low and middle income countries were included in the review. Research papers included in the review were assessed for quality using appropriate critical appraisal tools. In total 9 articles were included in the review; 5 of these related to health effects and 4 related to social effects of open defecation.

**Results:**

The review identified 4 overarching themes; Health Impacts of open defecation, Increased risk of sexual exploitation, Threat to women’s privacy and dignity and Psychosocial stressors linked to open defecation, which clearly present a serious situation of poor sanitation in rural communities of Lower-Middle Income Countries (LMICs). The findings of the review identified that open defecation promotes poor health in women with long-term negative effects on their psychosocial well-being, however it is a poorly researched topic.

**Conclusion:**

The health and social needs of women and girls remain largely unmet and often side-lined in circumstances where toilets in homes are not available. Further research is critically required to comprehend the generalizability of effects of open defecation on girls and women.

**Prospero registration:**

CRD42019119946. Registered 9 January 2019 .

## Background

Open defecation is defined as the practice of defecating in open fields, waterways and open trenches without any proper disposal of human excreta [1, 2]. The term “open defecation” is credited to the publications of Joint Monitoring Program (JMP) in 2008, a joint collaboration of World Health Organisation (WHO) and United Nations International Children’s Emergency Fund (UNICEF) to evaluate the global progress on water and sanitation goals. Open defecation is classified as unimproved sanitation [3]. Despite 15 years of conjunctive efforts under global action plans such as the Millennium Development Goals (MDGs), targets for improved sanitation were not met, resulting in 2.5 billion people not having access to improved sanitation facilities (flush latrine or pit latrine) and nearly 892 million of the total world’s population still practicing open defecation. As a result of this failure to successfully ensure basic sanitation it was once again highlighted as a key issue in the Sustainable Development Goals (SDGs). Number 6 of the Sustainable Development Goals (SDGs) is to “Ensure availability and sustainable management of water and sanitation for all”, where Target 6.2 aims by 2030 to achieve access to adequate and equitable sanitation and hygiene for all and end open defecation, paying special attention to the needs of women and girls and those in vulnerable situations [4]. Of those who still practice open defecation 90% of people reside in rural areas of three regions; sub-Saharan Africa, Central Asia and Southern Asia [3].

The health risks most researched in context of open defecation are those associated with human excrement linked infectious diseases [5]. Infected human excreta contain several harmful organisms that are associated with a number of health problems. Virtually, one gram of infected human excreta can contain a variety of microbes which includes 106 pathogenic viruses and infectious virions, 106–108 bacterial pathogens, 103 protozoan cysts and 10–104 helminth eggs [6]. Inappropriate human waste disposal also increases the risk of exposure to these pathogens which can pose significant health risks such as transferable infectious diseases, diarrhoea, typhoid and cholera, and viral infections [7]. WHO reports that 1.8 million people in low and middle income countries suffer from severe trachoma [8], a root cause of visual impairment which is transmitted via flies that breed on human excreta with a tendency to spread through eye discharge of infected person [9]. Likewise, more than 200 million people are infected with schistosomiasis (snail fever) worldwide [10], a chronic parasitic disease transmitted through human faeces to freshwater snails and the infection spread in humans when skin comes in contact with infection carrying snails or consumption of contaminated water and modulate their immune systems [11].

Open defecation is an issue that can affect everyone but women are often at more risk of experiencing violence and multiple health vulnerabilities [12]. Strunz et al. [13] identifies that women with poor sanitation facilities are more susceptible to hookworm infestation resulting in maternal anemia, which in turn is directly associated to adverse pregnancy outcomes [14]. Corburn and Hildebrand [15] also found that women with limited or no access to toilet predominantly suffered from diarrheal diseases, a leading cause of undernutrition among women during their reproductive age. The interaction between disease and undernutrition can further uphold vicious cycle of worsening infection and deterioration of women’s health, particularly in pregnant women [16]. However, Ziegelbauer et al. [17] argue that improved sanitation interventions can play constructive role in disease prevention, including diarrhoea and soil-transmitted infections.

Few researchers [15, 18, 19] claim that open defecation can lead to increased vulnerabilities to violence such as verbal, physical and sexual, affecting women physically and psychologically. Lack of household toileting facilities forces many women to travel long distances from their house to find private open places to defecate, manage their menstrual necessities which makes them vulnerable to these varying forms of violence [20]. Privacy considerations, cultural norms or religious practices also bound many women to wait until dawn or dusk so they would not be seen while fulfilling their basic need of defecating [21]. A case study by Nallari [22] illustrates the experience of young girls who defecate in vacant area beside their poor settlement in Bengaluru, India. The participants expressed both the fear of being exposed while passing the slums and the struggle of finding privacy. The heightened fear, shame and helplessness are common in the girls and women of Lower-Middle Income Countries where open defection still persists [18]. A key aspect of one’s human right is the right to physical security.

The United Nations (UN) [23] have challenged that sanitation has a major impact upon individual Human Rights, arguing that health implications linked to access to clean water, poor sanitation and open defecation are clear breaches of human rights. In addition, considering the implications for women and physical security in that women practicing open defecation are more at risk to violence, then the UN argue that a failure to address this at a national level is a form of gender discrimination a further violation of human rights [23]. However, the UN assert that sanitation has to be considered beyond the scope of just considering health, housing, education, work and gender equality but instead should be considered in terms of human dignity in that open defecation evokes feelings of vulnerability and shame and this infringement to human dignity should be considered a human rights issue.

This review addresses the following research question: To what extent does open defecation in lower-middle income countries present health and social effects on women and girls during their reproductive age? Most of the available literature indicates that the sanitation challenges are universal for girls and women in Lower-Middle Income Countries but their frequency and severity may vary in different settlements. The exploration of existing literature identified the paucity of evidence with no comprehensive systematic review being available to date. This is the first literature review to look at the association between open defection and its health and social outcomes on women and girls in an attempt to address the research gap in existing literature. The study objectives are: a) to provide insight into the breadth of available literature related to open defecation as a public health and social issue and b) to identify if there is an evidence gap regarding the health and social implications of open defecation on women and girls living in low and middle income countries. The findings of the study will have coherent implications for sanitation practitioners and scholars in low and middle income countries, particularly, those with keen interest in health and social outcomes of open defection. Therefore, the aim of this literature review is to investigate the health and social implications of open defecation on girls and women in Lower-Middle Income Countries.

## Methods

The study provides a systematic review of the published literature related to impacts of open defection that goes beyond the scope of addressing health outcomes and investigates the social outcomes associated with open defecation. The guidelines provided by Cochrane Collaborations were followed to conduct this review [24]. The selection of systematic literature review was adopted because the approach permits the identification, synthesis and appraisal of all the available research evidence in order to answer the research question in a robust and transparent manner [25].

### Data source

Electronic bibliographic databases were searched using predefined search terms for data source of primary literature. The databases used were EMBASE, MEDLINE, PsychoInfo, PubMed, Science Direct and Web of Science. Alternatively, a search was conducted by means of mySearch Bournemouth University, a search tool (iteration of EBSCO Discovery Service tool), to assist in simultaneous search of multiple electronic databases including the aforementioned.

### Search terms

Population, exposure and outcomes (PEO) tool is widely used as a concept map to provide an organised framework to list down the search terms articulated in the research question [26], therefore, an adopted version of PEO logic grid was constructed to identify the key search terms by using keywords, free-text words, index terms and synonyms selected with reference to the research question, electronic database and manual text mining from relevant published papers. Boolean operators and truncation were also used to connect the search terms (Table 2).

### Inclusion/exclusion criteria

The inclusion criteria and exclusion criteria is illustrated in Table 1 and Table 2. All articles were required to include analysis relevant to women, between the age of 13 and 50, who practice open defecation. Women during their reproductive age in rural communities have very similar household responsibilities and experience similar health and social challenges [27], hence, the age group was selected to draft a focused and consistent literature review. The review was restricted to low and middle income countries as they are the countries where the lack of available resources to ensure safe management of human excreta are highest [28]. The health implications highlighted in the search terms were left intentionally wide to cover all the potential diseases, while social outcomes of interest are chosen prudently based on prior literature reading. Moreover, no time limit was applied to the search databases in order to capture maximum key information for the review.

**Table 1:** Inclusion Exclusion Criteria

	Inclusion criteria	Exclusion criteria
Population	exclusively include analysis relevant to women between 13 and 50 years exposed to open defecation	All other population
Exposure	clear evidence of association between open defecation and its impacts on women’s physical, mental and social health, should include description of open defecation or inappropriate disposal of human excreta	study with no relevant analysis associating open defecation with health and social outcomes, no gender segregation
Place of study	low-income or developing countries (based upon world bank country classification)	developed countries
Setting	rural and remote areas	Urban areas
Time period	no time limit applied	–
Language	English or translated in English	All other languages
Study Design	empirical papers (primary or secondary data analysis), any primary study must consider ethical approval studies describing original articles published in a peer-reviewed journal	editorials, commentaries, policy documents, case study, opinion pieces

**Table 2:** Search Terms used in the PEO Framework

Population	Exposure	Outcome1	Outcome2
wom#n or girl* or female* or “adolescent girl*”		Health	Social
	Open defcat*	health or wellness or wellbeing or quality of life	“social effects”
		disease* or infection* or illness or sickness or diarrhea or cholera or typhoid or outbreak or “preventable diseases”	safety or danger or lynch or “psychosocial stress” or fear or embarrassment or rape or abuse or violence

*Refers to truncation of the search term

### Screening process

**Fig. 1:**
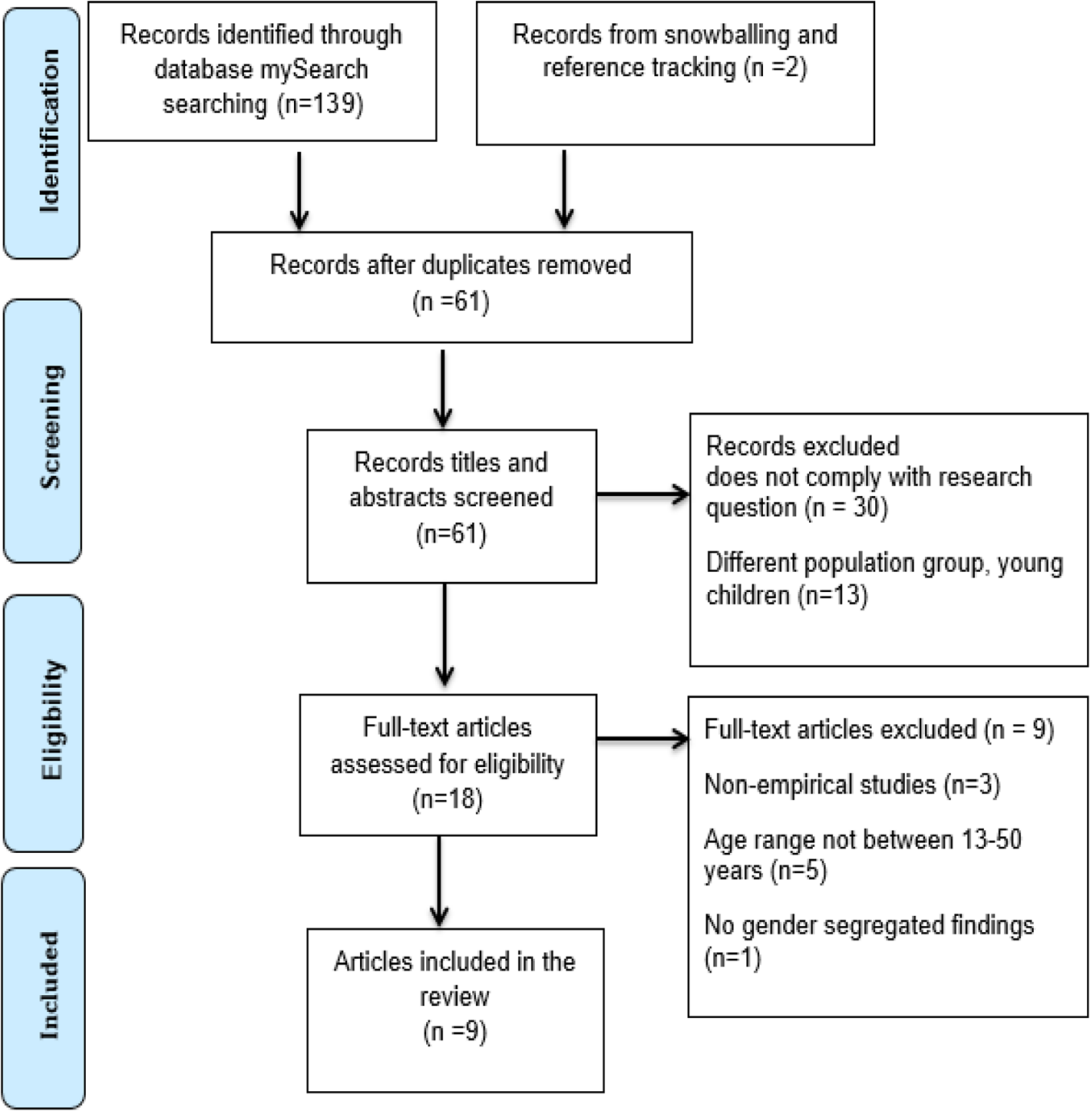
PRISMA Flow Process

The Preferred Reporting Items for Systematic Review and Meta-analysis (PRISMA) protocol was followed to facilitate the transparent reporting process of screening phase [29]. An initial screening of titles and abstracts was performed online to confirm that included studies broadly reflected the initial inclusion and exclusion criteria. When a title and abstract could not be excluded with confidence, the full text of the studies was obtained for full investigation using the predefined inclusion and exclusion criteria. Reference tracking is also an important technique for identifying studies published in masked journals [30], hence, the reference sections of all full text studies identified through the online database search were manually scour. 139 articles were identified using the search terms. An initial screening excluded 80 articles due to duplication. 2 additional articles were identified through reference tracking (snowballing). Screening of titles and abstracts using predefined inclusion criteria was performed for the remaining 61 articles. 30 articles were subsequently excluded as they were outside the scope of the investigation (focus on poor sanitation and lack of hygiene). 18 articles were not clearly excluded using title or abstracts, therefore, full text of articles were obtained for intensive screening against inclusion criteria, which further removed 9 articles because of the non-empirical research approach (*n* = 3), lack of gender segregated results (*n* = 1) and failure to meet the age range as per inclusion criteria (*n* = 5). The remaining 9 articles were included for the review; 5 of these related to health effects and 4 related to social effects of open defecation. Figure 1 illustrates the PRISMA flow process of included citations.

### Data extraction and analysis

A data extraction form was tailored to summarise the necessary information of retrieved studies which includes; geographic location, study design, study characteristics, methodological approach, findings and limitations. Data was summarized narratively and in tabular form for cross-study comparison, while meta-analysis was not performed due to the heterogeneity between included studies. Content analysis approach was applied, themes were developed by counting the frequency of recurrent important concepts across the retrieved studies, which offered a structured way to categorise the findings [31], summarised under separate headings in result section.

There is no single method to critically appraise the empirical evidence; nevertheless, the use of a standard checklist can be a reliable mean to ensure that each study is assessed according to its specific study design [32]. Therefore, the qualitative studies were evaluated against their validity and reliability by using Critical Appraisal Skills Programme (CASP) checklist [33], where quantitative studies were assessed for their credibility, transferability, dependability and conformability by using McMaster critical appraisal form [34]; and mixed method research articles were assessed by using an integrative combination of quantitative and qualitative appraisal tools to avoid any substantial bias i.e. mixed method appraisal tool (MMAT).

## Results

Four themes were identified; Health Impacts of open defecation, Increased risk of sexual exploitation, Threat to women’s privacy and dignity and Psychosocial stressors linked to open defecation.

### Study characteristics

Of all 9 retrieved studies, the most followed research method was quantitative approach which included 2 cross-sectional studies, 3 used secondary datasets and 1 experimental study. There were 2 qualitative studies; 1 used in-depth interviews and applied grounded theory methodology and the other was population-based cohort study (prospective). 1 study used mixed method research approach (survey, focus group discussion and interviews). All the studies were conducted in Lower-Middle Income Countries (7 India, 1 Kenya and 1 Cambodia) and published in English between 2009 and 2016 in peer-reviewed journals. Table 3 summarises the characteristics, findings and limitations of included studies.

**Table 3:** Summary of Included Studies

Authors, year and origin	Type and domain of study	Sample size (n)	Aim	Measurement of exposure and outcome	Findings and Recommendations	Limitations and Critical Appraisal
Jadhav et al. 2016 [38] India	Social: Quantitative (Secondary Data)	75,619 women (15–49 years)	Whether women’s lack of household sanitary facilities is associated with a higher likelihood of experiencing non-partner sexual violence (NPSV)	Based on two dichotomous questions Bivariate analysis for non-partner sexual violence and multivariate analysis to adjust participants’ demographic characteristics. Logistic regression used to conclude the results	Multiple results: 53% had access to toilets. 0.1% experienced sexual violence in past one year 8% had pit/latrine. 0.1% experienced non-partner sexual violence in past one year 39% use open defecation. 0.2% experienced sexual violence in past one year t-test confirmed the difference, sexual violence is twice as common among women using open defecation than it is among women using toilets (*p* < 0.05) 2.14 times more risk of sexual violence is experienced by women who use open defecation empirical research is required to investigate in-depth, improving social acceptability of toilet use and behavioural change is imperative,	Information from secondary data was not robust enough to explain the complex issue of open defecation in-depth; limited to past one year; findings potentially lack depth
Hirve et al. 2015 [39] India	Social: Mixed method approach	308 women 165 (13–17 years) 143 (18–35 years)	To examine the sources and extent of psychosocial stress linked with latrine use or open defecation by women at home, workplace or schools	Survey questionnaires: Likert scale. Electronically recorded, steps taken to avoid data entry errors, STATAv11 used to analyse the data, psychosocial stressors tested using t test and chi square test Focus Group Discussion and Key Informant Interview: Audio recorded in local language, translated in English used codes in MaxQDA software. A social scientist verified 10% of translated information as accurate and complete. Single and multiple codes are defined in detail. Coding verified by two researchers. Content analysis Free listing exercise: Smith’s solution	Clear evidence of association between various forms of stress and limited access to sanitation Toilet at home: 64% open defecators and 6% toilet users reported level of stress due to perceived lack of personal safety (*p* < 0.001) 44% open defecators and 3% toilet users reported stress level due to lack of privacy (*p* < 0.001) 47% open defecators and 5% toilet users reported stress due to insufficient cleanliness (p < 0.001) Toilet at workplace: 16% women open defecate at workplace- concerns about dirty toilets (29%) and unavailability of water (22%) but no concerns about access, personal safety or indignity reported during FGD **Toilet at schools:**6% open defecate, 82% toilet within yard, 13% toilet outside yard; common problem reported insufficient cleanliness and unavailability of water Overall education attainment difference in open defecators and toilet users Social and mental health impacts resulting from limited access to sanitation are equally important as physical health impacts of sanitation. Addressing sociocultural, environmental and behavioural barriers faced by women along with building toilets may empowered them by offering dignity and integrity	Potential bias of social desirability was identified as the personal security concerns faced by adolescent girls were voiced by the teachers of school instead of girls in a focus group discussion
Winter and Barchi 2015 [40] Kenya	Social: Quantitative (Secondary Data)	6191 women (15–49 years)	To study the relationship between violence against women and open defecation and role of social disorganisation in influencing this relationship	Content analysis: Stata/MP 13.1 statistical analysis 3 binary logistic regression, sensitivity analysis	Quantitative evidence of 40% greater odds of having experienced non partner sexual violence by women last 12 months (p < 0.05) Women practicing open defecation in highly disorganised communities have 13 times the odds of experiencing sexual violence (*p* < 0.01), open defecation in non-highly disorganised communities has no significant association	Limited information of past year Authors may have altered the scope of their analysis as secondary data provided limited information to facilitate their research question
Sahoo et al. 2015 [41] India	Social: Qualitative	56 women 14 below 25 years 14 newly married 15 pregnant 13 (25–45 years)	To study the psychological and social impacts of sanitation routines among women of reproductive age	Grounded theory research, 56 in-depth interviews, digitally recorded and transcribed using MaxQDA software	51 respondents faced environmental barriers to reach their site of defecation(fear of snake biting or other animals *n* = 32, infections *n* = 46, ghosts or supernatural forces *n* = 35) 51 participants had privacy concerns (newly married and pregnant women faced different social restrictions than adolescents i.e. approved time, place and support network needed before open defecation) 19 respondents encountered some sort of sexual violence (impact was severe for young girls) Psychosocial stress is mitigated by practicing open defecation in groups, performing sanitation related activities before dawn, limiting intake of food and liquids) Toilet structure is imperative but is not sufficient to address psychosocial stress, broader view of sanitation and open defecation is required	Based on interviews; reported incidents may be biased No confirmations of results, Cannot generalised
Janmohamed et al. 2016 [37] Cambodia	Health: Quantitative (Secondary Data)	*n* = 544 18–46 years	To investigate any relationship between household sanitation facility and Hb concentration, BMI and underweight among women in their first trimester of gestation	Multivariable linear and logistic regression models IBM SPSS software	Nearly 60% of study population practicing open defecation (non-improved sanitation facility) BMI and Hb concentrations found to be lower among women practicing open defecation than women using closed latrine (*p* = 0.01 and *p* = 0.001 respectively) Prevalence of moderate anemia was higher among women practicing open defecation (34% versus 23%) Cross-sectoral and integrated programs are required	Limited discussion of open defection as a contributing factor
Padhi et al. 2015 [35] India	Health: Population-based cohort study	670 pregnant women (18–48 years)	To quantify the risks of adverse pregnancy outcomes (APOs) i.e. event of preterm birth, low birth weight, abortions or miscarriages in relation to non-improved sanitation facility	Questionnaires and checklists Socio-demographic information obtained via face to face interviews Data analysed with STATA (version 13)	58.17% of had no access to latrine, Risk of preterm birth and low birth weight (p < 0.001) significantly increased for women who had no access to toilet and who used toilet occasionally Poverty correlated with lack of sanitation access Additional research required	Limited discussion of assessing the findings in context of literature evidence, lack of measurement of confounding risk factors (smoking, alcohol use, history of maternal health, etc.) for adverse pregnancy outcomes
Majumdar et al. 2009 [36] India	Health: Quantitative; Cross sectional study	193 pregnant women	To study the magnitude and correlates of hookworm infestation among pregnant women	Face to face interview, digital recording, laboratory specimen testing Chi-square test to analyse distributions of categorical variables	Open defecation is cofounding factor in prevalence of hookworm infestation among pregnant women Women defecating in open-fields were more infected with hookworm (24.3%) than women using sanitation latrine (6.8%) Focus on behavioural change is recommended	No age specified, Limited literature background, risk of selection bias as appropriate randomised sampling was not achieved (first 193 pregnant women were selected who visited antenatal clinic at that time)
Kotian et al. 2014 India	Health: Quantitative	Total sample size = 327 123 women all age group	To investigate the prevalence of intestinal parasitic infection in general population	Microbiological laboratory sample test,, multivariate data analysis	More women (17.07%) were infected with parasitic infection than men (8.33%) Prevalence of infection was higher in population practicing open defecation (22.69%) than people using latrines (11%) Interventions addressing early health education, proper waste disposal and safe drinking water were recommended	Single laboratory observation, Recruitment of participants from one hospital, cannot generalised, not transferable
Greenland et al. 2015 India	Health: Quantitative	1279 school children; 52% female (4–17 years)	To report the cases and intensity of soil-transmitted helminths	Sample test, survey (two-stage cluster sampling scheme); univariate and log binomial model, Stata 12 for analysis	52% participants were female, 63% of children practiced open defecation, no sex difference in the risk of infection, children over 12 years were likely to be more infected than others, children using open defecation were highly infected (48.9%) in comparison to children using toilets (13%)	sample size confined to girls between the age of 4 and 17 years

## Themes

### Health impacts of open defecation

The findings from the review demonstrate plausible evidence that open defecation has significant impact on health and well-being of women. Three studies [35–37] affirm the vulnerability of child-bearing women to open defecation which can be detrimental to both mother and the developing foetus. A prospective cohort of pregnant women study by Padhi et al. [35] which followed 670 pregnant women in their first trimester in rural India identified a statistically significant association (*p* < 0.001) between open defecation and adverse pregnancy outcomes such as preterm birth and low birth rate, however, the authors provided limited discussion of assessing their findings. Similarly, in west Bengal of India, Majumdar et al. [36] explored that open defecation is a confounding factor in the prevalence of hookworm infestation among pregnant women; in that pregnant women who defecate in open fields are at greater risk of hookworm infestation (24.3%) than those who use toilets (6.4%). The final study population may not be a generalised representation of target group as an appropriate randomised sampling was not achieved which reflects a potential risk of selection bias.

Risk of maternal complications increases with poor sanitation as it exacerbates the impacts of poor nutrition due to faecal-oral transmission of infections in pregnant women; a cluster-randomized efficacy trial by Janmohamed et al. [37] demonstrated that low body mass index (BMI) and low haemoglobin (Hb) level occurred in pregnant women of Cambodia who defecate in open in comparison to women with improved sanitation facility (closed pit latrine). Whilst a cross sectional survey by Greenland et al. [38], illustrates that children in rural settlement of India engaged in open defecation are more susceptible to soil-transmitted helminths (48.9%), intestinal infection transmitted through exposure of infective human faeces, than children who used toilets (13%). Since the sample size was confined to girls between the age of 4 and 17 years and no further age classification is provided, the study still validates that older girls (over 12 years) were more likely to be infected with soil-transmitted helminths than younger girls.

Lastly, Kotian et al. [39] found that people in Bihar State of India who used open defecation showed more positive results for parasitic infection, moreover, they also observed that the infection was more prevalent in female population (17.07%) than men (8.33%). They argued that the wide variation in prevalence of infections in study area can also be due to poor quality of available drinking water, higher engagement of women in livestock and agricultural management, inappropriate waste disposal practices or other environmental conditions; nevertheless, open defecation remains a major cause of water contamination, spreading of communicable diseases leading to immediate public health effects. However, the findings cannot be generalised to a larger population because the sample recruitment was restricted to the patients admitted to the hospital.

### Increased risk of sexual violence

Two studies [40, 41] focused on assessing the risk of non-partner sexual violence in relation to open defecation, whilst a qualitative Grounded Theory study [42] highlighted the experiences of sexual assaults and fear related to sexual violence among women after they leave their houses to defecate in open fields or near surroundings. Sahoo et al. [42] in Odisha, India also indicated that of all age groups the impact of sexual violence is severe among young unmarried girls. Likewise, Winters and Barchi’s [41] study analyzing cross-sectional data from the 2008–09 Kenya Demographic and Health Survey (DHS) identified that the risk of non-partner sexual violence increased to 40% among women who practiced open defecation than women who had the access to toilet facility (either in their household or shared toilet). Results from a logistic regression model [39] confirmed that there is a significant association between household sanitation facilities and non-partner sexual violence (*p* < 0.01) in India, concluding that non-partner sexual molestation incidents were two times more common among women who practiced open defecation than women who used toilets. Both quantitative studies [40, 41] analysed secondary data from national health surveys and had a fair number of participants (*n* = 75,619 and *n* = 6191), nonetheless, the authors may have restricted their scope of investigation to the available information which only presents the data of one year.

### Threat to women’s privacy and dignity

Two studies [42, 43] cited evidence in India to show how women who defecate in open fields experience several tangible threats to their privacy and dignity throughout the life. The findings from a focus group discussion [43] concluded that 44% of participants (*n* = 28) expressed the trauma of finding a suitable place to defecate in open fields and expressed indignity over holding off on defecation or urination when men or vehicles come within reach of their defecation site. Furthermore, the findings of in-depth interviews conducted in Odisha, India [42] presented that nearly all participants, 51 out of 56 women and girls, disproportionately expressed the fear of being watched or intruded by men in the absence of a toilet in their home.

### Psychosocial stressors linked to open defecation

The association of psychosocial stress to open defecation was strongly driven by two retrieved studies [42, 43]. A grounded theory study in India [42] explored a variety of psychosocial stress experienced by women during different life stages. The authors have listed those as; environmental (limited access, discomfort at defecation site, animals/insects) social (privacy, social restrictions and conflicts) and sexual stressors (peeping and sexual assaults). The most emphasized stressors were: searching for appropriate sites to defecate, travelling long distances, carrying water for cleaning, increased risk of insect or snake bites, fear of ghosts in dark and uncleanliness at site. They also illustrated that women preferred to travel in groups or accompanied by a relative when they need to defecate in open as a mitigating method because of the fear of being verbally, physically or sexually abused which were commonly reported by women who practiced open defection. Authors also discussed the influence of geographic settlement on women’s experience of sanitation related psychosocial stressors. Women from rural settings of Odisha India shared the highest number of their social stressors experiences; lack of facilities near house, social restrictions, insufficient privacy while defecating in open. Hirve et al. [43] reported that in Western rural India, psychosocial stress extends to concerns regarding personal safety as revealed by more than half of the participants (64%) and such stressors were the leading causes behind women feeling tensed, worried, depressed and irritated.

The impact is relatively severe for girls and women of reproductive age as they face an additional challenge of managing their menstruation while tackling the everyday need to defecation, however, Sahoo et al. [42] argues that the issue extend beyond young age and is significant throughout all life stages of women and it is imperative to acknowledge that the involvement of women in designing and placement of toilets stand the best chance of long-term success of reducing sanitation related psychosocial stresses among women.

## Discussion

The aim of this review was to assess the extent and strength of evidence regarding the social and health impacts of open defecation on young girls and women. The overarching themes emerged across the findings clearly present the daunting situation of poor sanitation in rural communities of Lower-Middle Income Countries. The findings also identified that open defecation promotes poor health in women and has long-term negative effects on their psychosocial well-being.

It is important to acknowledge limitations of the review. It was anticipated that by setting no time restriction to the search, using broad search terms, and using a number of different online databases, the occurrence of any literature selection bias would be prevented [44]. An additional step of reference tracking was taken to identify the relevant literature. Given the time constraint placed on this research, it was aimed to spread the search as wide as possible to yield literature evidence. Another limitation of this systematic review is that out 9 retrieved studies, 7 were conducted in India. This reflects inadequate representation of greater population of women exposed to open defection, also limiting the findings transferability to a different social or cultural setting in any other part of the world. A key challenge lay in the decision to not incorporate grey literature. In relatively under-researched topics such as open defecation there is a strong possibility that the most useful contextual information is captured by unpublished data such as fieldwork reports from non-governmental organisations or implementation of an intervention in an informal research. However, the potential effect arises from this bias is acknowledged and further exploration including grey literature can be useful to unfold a wider perspective of the issue in future.

All the health studies included in this review investigated open defecation under the umbrella of poor sanitation and were adjusted for multiple confounding variables, for example, availability of safe drinking water, means of wastewater treatment, practice and means of washing hands after defecation, geographic setting and socioeconomic conditions. Therefore, this review cannot present a strong conclusion regarding the association between open defecation and women’s health; however, it can be deduced that adverse effects on women’s health attributable to open defecation are significant and inappropriate human waste disposal increases the risk of contact to the infectious agents and likely to exacerbate exposure to health risks such as infectious diseases but the strength and route of infection can vary due to confounding factors of poor sanitation.

In terms of the associations between open defecation and psychosocial aspects of women’s health, from this review it appears that the adverse impacts of open defecation extend beyond the explicit consequences of infections and diseases. Women and girls are often disadvantaged because of different sociocultural and economic aspects that deny them equal rights with men [45]. Not only do they have different physical requirements from men but they also have an additional need for privacy and safety when it comes to their personal sanitation. Findings also revealed that activities like walking long distances in search of suitable site to defecate and carry water is an indication of additional burden which can be physically stressful and challenging to women, particularly for pregnant women. Given the social restrictions of conservative societies and social disorganization as confounding factors [46] that may bound women interaction with men they are not related to, they face deep shame and loss of personal dignity if indecently exposed to men outside their home**.** Despite the apparent inclusion of sanitation component in global action agendas, there is a scant high quality literature available associating social factors with open defecation, which has been a major obstacle in finding a meaningful relationship between open defecation and its social impacts on women. Available evidence is limited to the information extracted from secondary data source which is not robust enough to conclude that sexual violence occurs only in the context of practicing open defecation, however, the physical and verbal assault that women experience due to lack of household satiation facilitates can lead to increased fear, anxiety, sense of powerlessness, and shame.

Access to sanitation has been essential for human dignity, health and well-being [23]. Sanitation is also recognised as a human right in a resolution passed by UN General Assembly in 2010 [47]. The practice of open defecation poses serious health impacts and concern to women’s dignity when it transpires in densely populated areas, especially in rural communities with sanitation challenges [11]. The problem of open defecation largely persists in low and middle income countries, where lack of resources and limited national budget towards sanitation interventions can obstruct the path to provide adequate sanitation facilities to the entire population [48]. Not only the unfortunate situation of no access to toilet to the women is the infringement of basic human rights but it is also an indication of failure of the health and social care authorities who are accountable for ensuring adequate provision of fundamental sanitation facilities [49]. Many developing countries face similar challenge of translating gender-sensitive sanitation policies to practice [50]. Our study highlights the immediate need for more applied research in this area to reform national level sanitation programs and address policy implementation weaknesses in lower middle incomes countries.

Until now, public health research on open defecation has centred on its connection to various infectious diseases, with an emphasis on its association with ill health, particularly in children as they are more prone to diarrheal morbidity [51], but there is a limited body of evidence that pertain the research based literature on open defection and its impacts on women in lower middle income countries. The gap is more pronounced in context to exploring and preventing social outcomes associated with open defecation, including various forms of abuse and different psychosocial stress. Keeping all limitations in mind, it is perceived that the weight of the research that was identified in relation to health and social outcomes of open defecation lies in the construct of magnitude and prevalence of the exposure. The reviewed studies do not allow us to identify any approach which can be implemented to reduce open defecation in order to develop effective strategies to improving sanitation or designing national sanitation policies and programmes. Nevertheless, it is clear that open defecation is still one of the poorly researched topics that may have immediate and severe effects on women’s mental health and well-being.

## Conclusion

Open defecation is a taboo topic, masked in mystery, which is associated with far too many diseases, sufferings, indignity, social and psychological impacts that women have to endure as its outcomes. Many women and girls in Lower-Middle Income Countries are disproportionately affected by the lack of sanitation facilities which is a serious threat to their health and mental well-being, and struggle with managing their bodily function of defecation on daily basis, and are consistently compelled to adopt mitigating strategies. The health and social needs of women and girls remain largely unmet and often side-lined in circumstances where toilets in homes are not available. Further research is critically required to comprehend the generalisability of effects of open defecation on girls and women.

## Abbreviations

BMI: Body mass index
CASP: Critical Appraisal Skills Programme
Hb: Haemoglobin
JMP: Joint Monitoring Program
LMICs: Lower-Middle Income Countries
MDGs: Millennium Development Goals
MMAT: Mixed method appraisal tool
NPSV: Non-Partner Sexual Violence
PEO: Population, exposure and outcomes
PRISMA: The Preferred Reporting Items for Systematic Review and Meta-analysis
SDGs: Sustainable Development Goals
UN: United Nations
UNICEF: United Nations International Children’s Emergency Fund
WHO: World Health Organisation
